# Comparison of the specificity of rheumatoid factor detected by latex fixation with that of regulatory rheumatoid factor

**DOI:** 10.1002/jcla.23533

**Published:** 2020-08-12

**Authors:** Liubov Beduleva, Alexandr Sidorov, Kseniya Semenova, Igor Menshikov, Kseniya Fomina, Natalia Shklyaeva, Liliya Gilmanova

**Affiliations:** ^1^ Laboratory of Molecular and Cell Immunology Department of Immunology and Cell Biology Udmurt State University Izhevsk Russia; ^2^ Udmurt Federal Research Center UB RAS Izhevsk Russia

**Keywords:** conformers of IgG Fc fragments, latex fixation test, lyophilized IgG, pathological rheumatoid factor, regulatory rheumatoid factor, specificity

## Abstract

**Background:**

Rheumatoid factor (RF), originally defined as pathological autoantibodies to IgG that are detected in rheumatoid arthritis, turned out to be multi‐specific antibodies, some of which exhibit immunoregulatory properties. Recently, we identified a RF, the production of which confers resistance to experimental autoimmune diseases and is associated with the remission of autoimmune diseases. To differentiate the RF, we discovered from the one associated with rheumatic disease onset or progression and to reflect its immunoregulatory properties, we named it regulatory rheumatoid factor (regRF). Immunization with conformers of Fc fragments that expose regRF neoepitopes reduces collagen‐induced arthritis in rats. Certain information about the specificity of classical RF and regRF indicates that these populations may be one and the same. Therefore, the aim of this study was to determine whether there is a difference between the classical RF and regRF.

**Methods:**

Classical RF was measured in diseased blood by the latex fixation method, and regRF was detected by the agglutination of human IgG‐loaded tanned erythrocytes. Competitive analysis was used to determine the specificity of rheumatoid factors.

**Results:**

It was found that regRF and pathology‐associated RF constitute different antibody populations. Pathology‐associated RF is specific for lyophilized IgG. RegRF does not interact with IgG. RegRF is specific to conformers of IgG Fc fragments that have a reduced hinge. In latex‐positive rheumatoid arthritis sera, regRF may be present in addition to pathology‐associated RF. The latex fixation method detects both rheumatoid factor populations.

**Conclusion:**

RegRF and classical pathology‐associated RF have different specificity.

## Graphical abstract

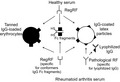

## 1 INTRODUCTION

Several decades have passed since rheumatoid factor (RF) was shown to be associated with rheumatoid arthritis, and later with certain other autoimmune diseases; however, the functions of rheumatoid factor and its role in the development of autoimmune disease remain a mystery. Numerous studies of the specificity and properties of rheumatoid factor have shown that rheumatoid factor is a population of antibodies that is heterogeneous in specificity and properties.^1^, ^2^, ^3^, ^4^ However, knowledge of rheumatoid factor diversity has not enabled us to solve the problem of the rheumatoid factor, but instead has deepened and expanded it.

Several years ago, while exploring the cause of the latent period in the formation of antibodies to collagen in collagen‐induced arthritis (CIA)‐resistant rats, we found that during that period (7‐14 days after collagen immunization), the level of rheumatoid factor in the rats’ blood increased. In rats sensitive to CIA, antibodies to collagen had already appeared on day 7 after immunization, and no RF production was observed during this time.^5^ We also observed a similar association between RF production preceding the production of antibodies to the inducer antigen and resistance to autoimmune disease in models of experimental autoimmune rat encephalomyelitis (EAE) and rat atherosclerosis induced by immunization with native lipoproteins.^6^ In addition, we noticed a connection between RF production and the remission of CIA and EAE.^6^ To detect RF, we used the method of agglutination of tanned erythrocytes loaded with homologous IgG. A study of the specificity of the rheumatoid factor detected by this method showed that it is a species of anti‐idiotypic antibodies of varying specificity that are united by the presence of a common paratope specific for the neoepitopes of conformers of IgG Fc fragments.^6^, ^7^, ^8^ Immunization with conformers of IgG Fc fragments carrying neoepitopes recognized by rheumatoid factor reduces collagen‐induced arthritis in rats.^7^ Therefore, all of the information we obtained about the rheumatoid factor detected by the agglutination of tanned IgG‐loaded erythrocytes pointed to the regulatory properties of RF. We suggested that the rheumatoid factor we were studying represents an independent population that differs from the classical rheumatoid factor associated with rheumatoid arthritis, and we named it “regulatory rheumatoid factor”.^7^

At the same time, certain findings regarding the specificity of classical rheumatoid factor and regulatory rheumatoid factor point to the possibility that these antibody populations are one and the same. In particular, the classical RF associated with rheumatoid arthritis is specific for cryptic epitopes that are absent from the native IgG molecule and appear as a result of changes in its conformation upon interaction with antigen or heat or chemical treatment.^8^ RegRF epitopes are also neoepitopes.^7^, ^8^ They are absent from IgG and appear on its papain Fc fragments as a result of the reduction of hinge disulfide bonds.^7^, ^8^

Moore and Dorner,^10^ in a review article, summarize the experimental evidence of double‐reactivity polyclonal RFs obtained from rheumatoid arthritis patients. RFs have topographically distinct binding sites, one for IgG, the other for the various targets. The regRF we identified is also a double‐specificity antibody. Each regRF molecule has a shared paratope that recognizes the unique idiotype of antibody, and a particular paratope specific for the neoepitopes of the conformers of IgG Fc fragments.^6^, ^7^, ^8^

The aim of this study was to find out whether the specificity of classical rheumatoid factor detected in the blood of patients differs from that of regulatory rheumatoid factor. To detect classical rheumatoid factor in the blood, we used the latex fixation test (LFT), which is widely used in clinical diagnostics and remains the standard to this day.

## 2 MATERIALS AND METHODS

### 2.1 Patient sera and donor sera

Sera were obtained from patients at Rezhevskaya Central Regional Hospital (Rezh, Russia), where patients with rheumatoid arthritis or symptoms of joint pain and swelling had come for help and been prescribed laboratory testing, including an assay of rheumatoid factor. Healthy human sera were obtained at the Republic Blood Transfusion Station (Izhevsk, Russia). The authors of this article had no contact with patients or donors and were not involved in the collection of sera. The sera were used anonymously.

### 2.2 Lyophilized IgG

Normal lyophilized IgG (Equitech‐Bio) was used to prepare an agglutination test system for detecting regRF and was used in a competitive analysis.

### 2.3 Native IgG

IgG that was considered native was isolated from the blood plasma of healthy donors by precipitation with ammonium sulfate and purified by size‐exclusion chromatography. The native IgG was used in a competitive assay.

### 2.4 Measurement of RF by the latex fixation method

The RF direct latex test (VedaLab) was used to detect rheumatoid factor in diseased sera. The agglutination activity of the sera was scored from “+++” (or 3) to “−” (or 0). In some samples, the titer was determined.

### 2.5 Measurement of regRF

The regRF titer was determined in an agglutination test using human IgG‐loaded tanned human erythrocytes. For this, group O human erythrocytes (Republic Blood Transfusion Station) were fixed with 1% glutaric dialdehyde. The erythrocytes were washed and then incubated for 10 minutes with the same amount of tannin solution in PBS at RT. For sensitization, 4 mL of PBS (pH 6.4), 1 mL of a solution of 0.5 mg/mL normal IgG from human serum (Equitech‐Bio) in 0.9% NaCl, and 70 μL of the pelleted tanned erythrocytes were mixed. Incubation lasted for 20 minutes at RT; the erythrocytes were washed with 0.9% PBS containing 0.2% BSA. Twofold serial dilutions of serum were prepared and put into wells in aliquots of 50 μL. The same amount of human IgG‐loaded 1.5% erythrocyte suspension was then added. Agglutination results were read after 3 hours.

### 2.6 Papain fragmentation of human IgG and separation of Fc fragments

To produce papain Fc fragments of IgG, we used the limited proteolysis method. Human IgG (Equitech‐Bio, Kerrville, TX, USA) was digested by papain at a ratio of substrate to enzyme of 100:4 (w/w) in the presence of 0.03 mol/L EDTA and 0.015 mol/L sodium phosphate buffer with 0.15 mol/L NaCl, pH 8.0, for 40 minutes at 37°C. The mixture was separated into two fractions by gel filtration on Sephacryl S‐100 (26 × 400) with 0.015 mol/L sodium phosphate buffer, pH 8.0. The first fraction was reduced by treatment with 0.014 mol/L β − mercaptoethanol for 2 hours and separated into two fractions by gel filtration on Sephacryl S‐100 (26 × 400) with 0.015 mol/L sodium phosphate buffer, pH 8.0. The first fraction contains intact IgG; the second fraction contains IgG fragments. Human IgG fragments were applied to protein G‐Sepharose. Some of the Fab fragments were eluted in free volume. The mixture of sorbed Fc and Fab fragments was eluted with 0.1 mol/L glycine‐HCl, pH 2.7, and then applied to protein A‐Sepharose. The Fc fragments were eluted with 0.1 mol/L glycine‐HCl, pH 2.7. Fab fragments were eluted in the free volume. AKTA purifier UPC (GE Healthcare) and Spectrophotometer Genesys 10S UV‐Vis (Thermo Fisher Scientific, Inc. U.S) were provided by the Center for the Collective Use of Scientific Equipment, UdSU.

### 2.7 Electrophoretic analysis of IgG Fc fragments

IgG Fc fragments were studied in 3%‐15% gradient polyacrylamide gel in dissociating and dissociating/reducing conditions.

### 2.8 Detection of neoepitopes recognized by regRF on human IgG Fc fragments

The presence of neoepitopes recognized by regRF on human IgG Fc fragments was tested in the binding inhibition reaction of regRF‐containing human healthy serum with human IgG‐loaded tanned erythrocytes. RegRF‐containing serum was titered from 1:2 to 1:2048 in increments of 2. The samples of Fc fragments (10 μg in a volume of 25 μL) were mixed with each dilution of regRF‐containing serum (25 μL). Next, human IgG‐loaded tanned erythrocytes (50 µL, 1.5% suspension) were added. Addition of IgG (30 μg in a volume of 25 μL) or PBS (25 μL) instead of Fc fragments was used as a control.

### 2.9 Competitive inhibition of latex fixation induced by rheumatoid arthritis serum

The RF titer in rheumatoid arthritis serum was determined by latex fixation. Next, 25 μL of a solution of Fc fragments (10 μg per well) or 25 μL of normal lyophilized IgG (30 μg per well) or 25 μL of native IgG (30 μg per well) was added to dilutions of 25 μL of serum, including the titer dilution. As a control, 25 μL of PBS was added in place of the proteins. The solutions were incubated for 1 hour at 37°C and then mixed with 50 μL of a suspension of IgG‐coated latex particles (RF latex test). The reaction was read after 2 minutes.

### 2.10 Competitive inhibition of tanned IgG‐loaded erythrocyte agglutination induced by rheumatoid arthritis serum or healthy human serum

To a series of serial dilutions, including the titer dilution, of rheumatoid arthritis or healthy serum (25 μL), an equal volume of conformers of IgG Fc fragments exhibiting regRF neoepitopes (10 μg per well) or normal lyophilized IgG (30 μg per well) or native IgG (30 μg per well) was added. Next, 50 μL of a 1.5% suspension of tanned IgG‐loaded erythrocytes was added to this mixture. The mixture was incubated for 3 hours at 37°C. As a control, 25 μL of PBS was added in place of the proteins.

### 2.11 RegRF depletion from the rheumatoid arthritis sera

Latex‐positive rheumatoid arthritis serum taken in a volume of 100 μL was mixed with 100 μL of a 7% suspension of tanned IgG‐loaded erythrocytes. The mixture was incubated for 1 hour at 37°C. The erythrocytes were separated by centrifugation. The resulting supernatant was used. Serum regRF levels before and after depletion were compared by agglutination of tanned IgG‐loaded erythrocytes.

### 2.12 Statistical analysis of the data

The significance of differences was assessed by the Wilcoxon matched pairs test or *t* test.

## 3 RESULTS

### 3.1 RegRF in latex‐positive diseased serum

**Figure 1:**
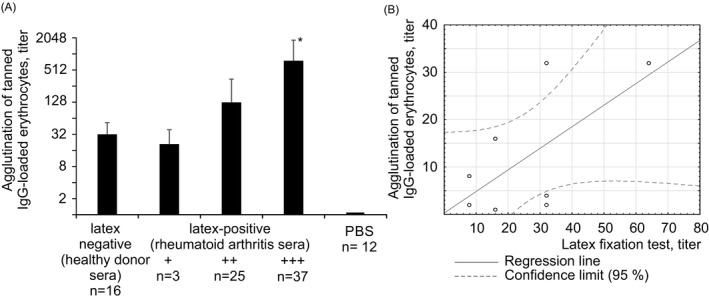
Agglutination of tanned IgG‐loaded erythrocytes and IgG‐coated latex particles by patient sera. A, Agglutination of tanned IgG‐loaded erythrocytes by healthy sera and latex‐positive patient sera. The results are presented as means ± SD. *—Statistically significant in relation to group “++,” *P* ≤ .05 (*t* test). B, Correlation analysis

Sera taken from patients diagnosed with rheumatoid arthritis as well as from patients being examined after presenting with complaints of joint pain and swelling were tested for rheumatoid factor by the latex fixation method. Sera found to be latex‐positive were selected. Most (77%) of the sera selected had been obtained from patients with an established rheumatoid arthritis diagnosis, made using EULAR diagnostic criteria. The remaining latex‐positive sera were from patients complaining of joint pain who did not yet have a diagnosis. Hereinafter, the latex‐positive sera obtained from both rheumatoid arthritis patients and from as‐yet undiagnosed patients are referred to as latex‐positive diseased sera (Figure 1A). In the experiments where sera only from diagnosed rheumatoid arthritis patients were used, the sera are referred to as “rheumatoid arthritis sera.”

Latex‐positive diseased sera and latex‐negative healthy sera were studied by agglutination of tanned IgG‐loaded erythrocytes. It was found that both diseased and healthy sera cause agglutination of tanned IgG‐loaded erythrocytes. Comparison of the intensity of the latex fixation reaction with that of the tanned IgG‐loaded erythrocyte agglutination reaction induced by diseased sera showed that the higher the activity of diseased sera in the latex test, the higher the average RF level detected in the sera by the tanned IgG‐loaded erythrocyte agglutination method (Figure 1A).

However, individual analysis showed that in diseased sera assigned to the same group based on the intensity of the latex fixation they induced, the titers detected by the agglutination of tanned IgG‐loaded erythrocytes vary significantly. In the +++ group (latex test), about 40% of sera had a relatively low titer in the tanned loaded IgG erythrocyte agglutination reaction (from 1:32 to 1:128), comparable to the titer of sera from the “+” and “++” groups (latex test). This suggests that in diseased serum, in addition to the RF detected by tanned IgG‐loaded erythrocyte agglutination, there are other antibodies detectable only by latex fixation.

In some latex‐positive diseased sera, the RF titer was measured by latex fixation and then compared with the RF level measured by the agglutination of tanned IgG‐loaded erythrocytes. Correlation analysis did not reveal a significant relationship between the RF level revealed by the agglutination of tanned IgG‐loaded erythrocytes and the RF level revealed by latex fixation (k = 0.64) (Figure 1B). Thus, latex‐positive diseased sera induce agglutination of tanned IgG‐loaded erythrocytes. However, the RF level determined by the method of agglutination of tanned IgG‐loaded erythrocytes does not correlate with the RF level detected by latex fixation.

In order to determine the specificity of antibodies detected in patient sera by agglutination of tanned IgG‐loaded erythrocytes, the effect of conformers of human IgG Fc fragments carrying neoepitopes recognized by regulatory rheumatoid factor on the binding of rheumatoid arthritis sera to tanned IgG‐loaded erythrocytes was studied. The presence of regRF neoepitopes on IgG Fc fragments was previously established by the ability of IgG Fc fragments to inhibit agglutination of tanned IgG‐loaded erythrocytes induced by healthy human serum.

**Figure 2:**
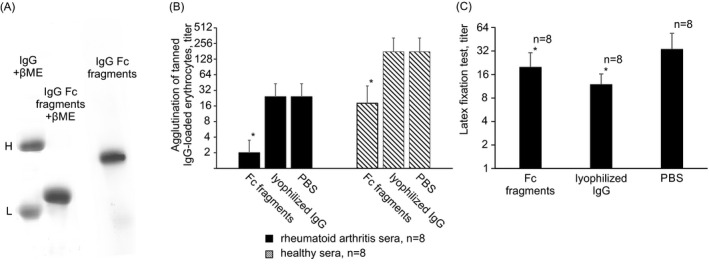
Inhibition of binding of healthy or rheumatoid arthritis sera with IgG‐coated tanned erythrocyte and latex particles by IgG Fc fragments carrying regRF neoepitopes and Iyophilized IgG. A, PAGE of papain IgG Fc fragments. 3%‐15% gel. βME = beta‐mercaptoethanol. B, Inhibition of the IgG‐coated tanned erythrocyte agglutination. *—Statistically significant in relation to PBS, *P* < .05 (Wilcoxon matched pairs test). C, Inhibition of latex fixation reaction. *—Statistically significant in relation to PBS, *P* < .05 (Wilcoxon matched pairs test)

Conformers of human IgG Fc fragments (Figure 2A) carrying neoepitopes recognized by regulatory rheumatoid factor (Figure 2B) were found to completely inhibit the tanned IgG‐loaded erythrocyte agglutination reaction induced by latex‐positive rheumatoid arthritis sera (Figure 2B).

We have previously shown that the binding of healthy human sera to tanned IgG‐loaded erythrocytes does not change in the presence of IgG.^7^, ^8^ On this basis, we concluded that IgG does not carry neoepitopes recognized by regulatory rheumatoid factor.^7^, ^8^ In the course of this study, we found that lyophilized (Figure 2B) and non‐lyophilized IgG preparations also did not affect the binding of rheumatoid arthritis sera to tanned IgG‐loaded erythrocytes.

In sum, in latex‐positive rheumatoid arthritis sera there are antibodies that cause agglutination of tanned IgG‐loaded erythrocytes. The binding of these antibodies is inhibited by conformers of Fc fragments carrying neoepitopes recognized by regulatory rheumatoid factor, but does not change with exposure to IgG, which does not carry these neoepitopes. Therefore, regulatory rheumatoid factor is present in latex‐positive rheumatoid arthritis sera.

Rheumatoid arthritis sera, unlike healthy sera, test positive in the latex fixation test. Therefore, the question arises, what drives their reactivity to IgG particles layered on latex? Is it induced by high regRF or the presence of other antibodies?

### 3.2 Comparison of the specificity of RF detected by latex fixation and that of regRF detected by tanned loaded IgG erythrocyte agglutination in rheumatoid arthritis serum

As shown above, the tanned IgG‐loaded erythrocyte agglutination reaction induced by rheumatoid arthritis serum is inhibited by the conformers of IgG Fc fragments, but not by IgG. Therefore, we tested whether these antigens will affect the binding of the same rheumatoid arthritis sera to latex particles. If the antigens behave in the same way, then the regRF and RF detected by the latex fixation method are the same antibodies.

We found that conformers of IgG Fc fragments carrying neoepitopes recognized by regulatory rheumatoid factor inhibit the binding of rheumatoid arthritis sera to latex particles (Figure 2C). However, IgG (lyophilized preparation) also inhibits the latex fixation reaction induced by rheumatoid arthritis sera (Figure 2C). The ability of IgG to inhibit the binding of rheumatoid arthritis sera to latex particles indicates that the rheumatoid arthritis serum contains RF that is specific for IgG and is detected by latex fixation. Since the regRF detected by the agglutination method is not specific for whole IgG (lyophilized preparation), but the RF detected by latex fixation is specific for it, then the regRF detected in rheumatoid arthritis serum and the RF specific for IgG detected by latex fixation are antibodies of different specificity.

### 3.3 Does the latex test detect regulatory rheumatoid factor?

How Fc conformers carrying regRF neoepitopes inhibit the latex fixation reaction induced by rheumatoid arthritis serum remains an open question. Either the latex particles coated with IgG carry neoepitopes specific for regRF and then the latex test reveals regRF in addition to RF specific for IgG, or IgG Fc fragments inhibit rheumatoid arthritis serum binding to the latex because they carry epitopes located on the Fc region of whole IgG. If the latter is true, then the latex fixation method does not reveal regRF, and the reason that conformers of Fc fragments inhibit latex fixation is that they bear epitopes of the whole IgG molecule.

To determine which of these assumptions is true, regRF was depleted from rheumatoid arthritis serum using tanned IgG‐loaded erythrocytes. If Fc fragments carry IgG epitopes, they will retain the ability to inhibit the binding of depleted serum to latex particles. If after depletion the ability of IgG Fc fragments to inhibit latex agglutination is lost, then the latex test reveals regRF.

**Figure 3:**
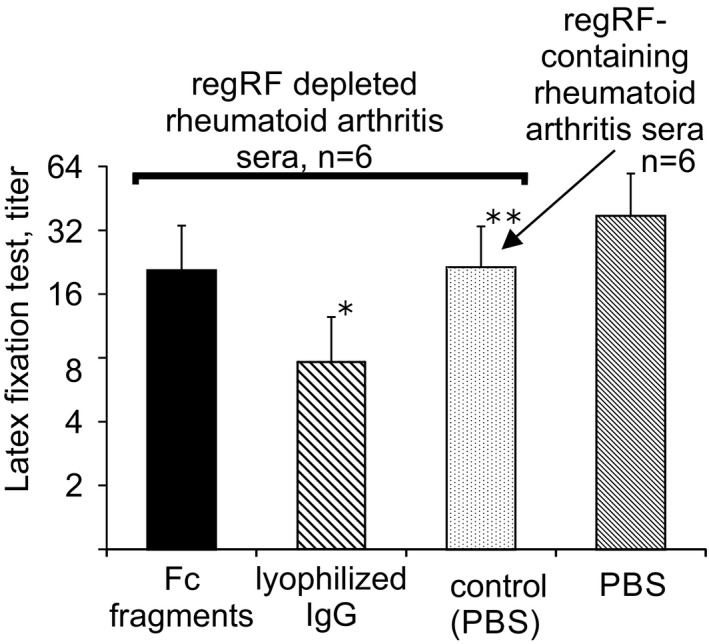
Influence of lyophilized IgG and IgG Fc fragments exhibiting regRF neoepitopes on binding of regRF‐depleted rheumatoid arthritis sera to IgG‐coated latex particles. *—Statistically significant in relation to control (PBS), *P* = .03 (Wilcoxon matched pairs test). **—Statistically significant in relation to regRF‐containing rheumatoid arthritis sera, *P* = .04 (Wilcoxon matched pairs test)

It was found that after regRF was depleted from rheumatoid arthritis sera, IgG Fc fragments exhibiting regRF neoepitopes lost the ability to inhibit the binding of rheumatoid arthritis serum to latex particles (Figure 3). IgG continued to inhibit the binding of regRF‐depleted rheumatoid arthritis serum to latex particles (Figure 3). Consequently, Fc fragments carrying regRF neoepitopes inhibit the binding of rheumatoid arthritis serum to latex particles not because they carry epitopes of whole IgG, but because this test reveals regRF.

Figure 3 shows that after regRF depletion, serum titers decreased in the latex fixation test. Since regRF is detected by latex fixation, the decrease in serum titer in the latex fixation test after regRF depletion is understandable. Thus, the RF latex test reveals two types of rheumatoid factor: regRF and RF specific for IgG.

### 3.4 RF specific for IgG detected in rheumatoid arthritis sera

**Figure 4:**
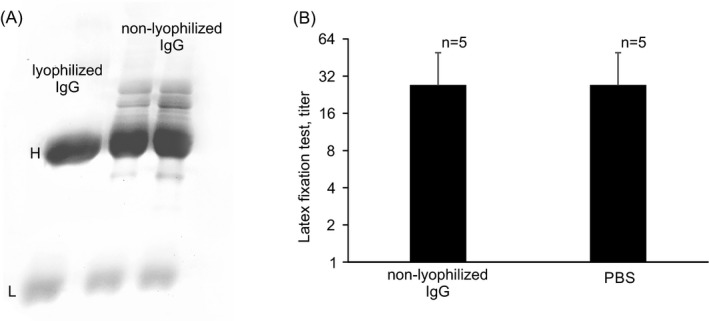
Influence of non‐lyophilized IgG on binding of rheumatoid arthritis sera to IgG‐coated latex particles. A, PAGE of non‐lyophilized IgG and lyophilized IgG. 3%‐15% gel. B, Latex fixation reaction

The IgG used to inhibit serum binding to latex particles, and tanned IgG‐loaded erythrocytes was a commercial preparation of lyophilized IgG. We considered this IgG to be native; however, its specificity for pathological RF, which does not react with native IgG,^6^ suggests that the structure of this IgG is different from the native one. Therefore, we isolated IgG from the blood plasma of healthy donors by precipitation with ammonium sulfate and subsequent size‐exclusion chromatography and did not lyophilize it. Non‐lyophilized IgG (Figure 4A), unlike lyophilized IgG, was unable to inhibit the binding of rheumatoid arthritis serum to latex particles (Figure 4B). Thus, the RF detected in rheumatoid arthritis serum by latex fixation is specific for neoepitopes that appear on IgG during lyophilization.

## 4 DISCUSSION

Numerous studies of RF have shown that RF is multi‐specific and that the RFs include regulatory RF and pathological RF. However, the specificity of pathology‐associated RF and of regulatory rheumatoid factor as well as the methods that can be used to detect them remained unclear.^11^ The aim of this study was to compare the specificity of the regulatory rheumatoid factor detected in the blood of healthy donors by agglutination of tanned IgG‐loaded erythrocytes with that of the rheumatoid factor detected in the blood of rheumatoid arthritis and rheumatoid disease patients using latex fixation.

The specificity of the regulatory rheumatoid factor detected by the agglutination of tanned IgG‐loaded erythrocytes was previously elucidated. regRF is specific for the neoepitopes of IgG Fc fragments that are exposed on Fc containing a reduced hinge.^8^ RegRF does not interact with IgG.^8^ In the course of this study, we demonstrated that pathology‐associated RF detected by direct latex fixation is specific for IgG epitopes that appear on IgG upon lyophilization. Pathology‐associated RF does not interact with non‐lyophilized IgG or with non‐lyophilized IgG Fc fragments. Lyophilization is known to lead to conformational changes in proteins. In particular, the lyophilization procedure leads to a decrease in α‐helix and random structure and an increase in β‐sheet structure.^12^ In the presence of residual moisture, freeze‐dried proteins can undergo disulfide interchange and other reactions.^13^ Therefore, the data we obtained on the specificity of RF detected in the blood of rheumatoid arthritis patients are consistent with the data that the pathological RF is specific to epitopes that are hidden in the IgG structure (cryptic epitopes) and only become exposed by conformational changes, which may occur upon antigen binding in vivo or in vitro, upon immobilization on surfaces (eg, the hydrophobic polystyrene surface used in routine ELISA for RFs), or under certain physico‐chemical conditions (eg, elevated temperature).^9^

Thus, the regulatory rheumatoid factor detected in the blood of healthy donors by agglutination of tanned IgG‐loaded erythrocytes and the pathology‐associated rheumatoid factor detected by latex fixation differ in their specificity.

The latex fixation method reveals in rheumatoid arthritis serum not only the pathology‐associated RF specific for lyophilized IgG, but also regulatory rheumatoid factor.

Since the latex fixation test reveals both regulatory RF and pathology‐associated RF, when assaying RF by the latex fixation method, it is impossible to ascertain how much regRF and how much pathology‐associated RF the diseased blood contains. Agglutination of tanned, IgG‐loaded erythrocytes induced by both healthy and diseased serum is not inhibited by immunoglobulin G, to which the pathology‐associated RF is specific. Therefore, the tanned IgG‐loaded erythrocyte agglutination test selectively detects regulatory rheumatoid factor. Future studies should determine the utility of assaying regRF levels for patient condition assessment, treatment effectiveness or prognosis.

## 5 CONCLUSION

RegRF and pathology‐associated RF are populations of rheumatoid factor that have different specificity. RegRF is specific for conformers IgG Fc fragments that have a reduced hinge, but not for IgG. Pathology‐associated RF is specific for epitopes appearing on lyophilized IgG. In latex‐positive rheumatoid arthritis sera, regulatory rheumatoid factor may be present in addition to pathology‐associated RF. The latex fixation method detects both rheumatoid factor populations. The tanned IgG‐loaded erythrocyte agglutination test selectively detects regulatory rheumatoid factor.

## AUTHOR CONTRIBUTIONS

LB involved in conceptualization, methodology, investigation, writing, validation, and supervision. IM involved in conceptualization, writing, reviewing, and editing. AS, KS, KF, NS, and LG involved in investigation.

## ETHICAL APPROVAL

This study was performed in line with the principles of the Declaration of Helsinki. Data were de‐identified. Approval was granted by the Bioethics Committee of Udmurt State University (Date 30.01.2020/No 2001).

## Data Availability

Data may be obtained from a third party and are not publicly available.
